# Water Resistant Cellulose – Titanium Dioxide Composites for Photocatalysis

**DOI:** 10.1038/s41598-018-20569-w

**Published:** 2018-02-02

**Authors:** Uthpala M. Garusinghe, Vikram S. Raghuwanshi, Warren Batchelor, Gil Garnier

**Affiliations:** BioResource Processing Research Institute of Australia (BioPRIA), Department of Chemical Engineering, Monash University, Clayton, 3800 Victoria Australia

## Abstract

Novel water resistant photocatalytic composites of microfibrillated cellulose (MFC)—polyamide-amine-epichlorohydrin (PAE)—TiO_2_ nanoparticles (NPs) were prepared by a simple two-step mixing process. The composites produced are flexible, uniform, reproducible and reusable; they can readily be removed from the pollutant once used. Small amount of TiO_2_ NPs are required for the loaded composites to exhibit a remarkable photocatalytic activity which is quantified here as achieving at least 95% of methyl orange degradation under 150 min of UV light irradiation for the composite with best combination. The cellulose network combined with PAE strongly retains NPs and hinders their release in the environment. PAE dosage (10 and 50 mg/g MFC) controls the NP retention in the cellulose fibrous matrix. As TiO_2_ content increases, the photocatalytic activity of the composites levels off to a constant; this is reached at 2wt% TiO_2_ NPs for 10 mg/g PAE and 20wt% for 50 mg/g PAE. SEM and SAXS analysis confirms the uniform distribution of NPs and their formation of aggregates in the cellulose fibre network. These economical and water resistant photocatalytic paper composites made by a simple, robust and easily scalable process are ideal for applications such as waste water treatment where efficiency, reusability and recyclability are important.

## Introduction

Inorganic nanoparticles-polymer composites have recently gained much attention for engineering functional materials and interfaces. Metals and metal oxides nanoparticles (NPs) such as TiO_2_^1^, Au^2^ and Fe_2_O_3_^3^ are distinct materials with size dependent properties in photocatalysis and photoelectronics applications^4^. Among those, TiO_2_ NPs are low cost material for industrial applications in photocatalysis, photochemical hydrogen production^5^, water purification and solar energy conversion^6,7^. There are many recent publications related to TiO_2_ photocatalysis^8–16^.

In 1972, Fujishima and Honda discovered photocatalysis with TiO_2_ NPs^17^. Anatase TiO_2_ gives high oxidizing power when irradiated by UV light, which has generated tremendous interest thanks to its low cost, high chemical stability and low toxicity^18–20^. Optical excitation with energy exceeding TiO_2_ band gap energy results in the formation of conduction band electrons and valence band holes. Both are powerful reductants and oxidants^21^. Hydroxyl radicals produced in TiO_2_ can be used to convert many organic compounds to CO_2_ and H_2_O. Therefore, TiO_2_ has been used to decompose various environmental pollutants^22,23^. The size and the length scale of these NPs play a major role in its properties and applications^24^.

Nano scale TiO_2_ (1–100 nm) possesses high surface area and shows enhanced photocatalytic activity^24^. However, TiO_2_ tendency to form agglomerates can significantly decrease its activity^25–27^. Using bare TiO_2_ NPs in water treatment has issues in their collection and poses uncontrolled NPs release as an environmental danger^28–31^. Incorporating TiO_2_ NPs directly into a matrix combines the advantages of NPs stability and retention; this enables water treatment without risk of NPs leaching or contamination^32^.

Previously, researchers have engineered composites with TiO_2_ NPs embedded in different networks such as silicon, carbon fibre, cellulose fibre and polypropylene/clay^33–36^. However, these composites have issues either in retention of NPs, are expensive to produce, difficult to recycle, non-biocompatible, lack of reusability or are not showing effective or controlled photocatalytic activity. There is a lack of fundamental understanding of the retention, dispersion and aggregation of NPs for the controlled photocatalysis activity of composites.

Many strategies have been explored for retaining inorganic NPs in sustainable material networks^37–39^. In this category, microfibrillated cellulose (MFC) is a low cost, biodegradable and recyclable natural fibrous matrix having high specific strength and surface area promising good NPs integration^31,40,41^. MFC is more stable in aqueous environments than conventional wood fibres^42,43^. Previously, many methods to produce and characterise MFC-NPs composites with high NP loadings (80 wt%) and controlled nanostructures were reported^38,44,45^.

We raise the hypothesis that the content and the aggregation state of TiO_2_ in MFC composites control their photocatalytic activity when exposed to UV light. Our proposed methodology is to disperse TiO_2_ NPs in a MFC network with controlled retention, distribution and agglomeration, while keeping the wet strength of the produced composites. These parameters are believed to be important variables for optimizing the photocatalytic activity of composites. Therefore, a material which serves as both wet strength and NP’s retention aid is required. Polyamide-amine epychlorohydrin (PAE) is a widely used wet-strength agent in the tissue and packaging industries. Its wet strength develops primarily by ester bond formation between the azetidinium groups of PAE with the carboxyl groups of the bleached cellulose fibres and is achieved during the drying process^46^.

In this study, MFC is investigated as TiO_2_ NPs carrier to produce water resistant fibrous composites of varying NPs loading. Here, PAE is used both as wet strength agent to consolidate the MFC structure and as a retention aid for the TiO_2_ NPs. Photocatalytic activity of composites with different NPs and PAE dosages is monitored by measuring the degradation kinetics of Methyl Orange (MO) solutions by UV irradiation. By changing the PAE concentration, we aim to vary the TiO_2_ aggregation state. Scanning electron microscopy (SEM) and Small angle X-ray Scattering (SAXS) measurements are used to quantify TiO_2_ NP distribution in the composites and are analysed in terms of PAE content. Our objective is to produce bio-compatible and reusable TiO_2_ NPs/MFC composites of controlled photocatalysis. Further, we aim at exploring TiO_2_ catalytic activity in terms of NPs distribution and aggregation state controlled with PAE dosage.

## Results

**Figure 1:**
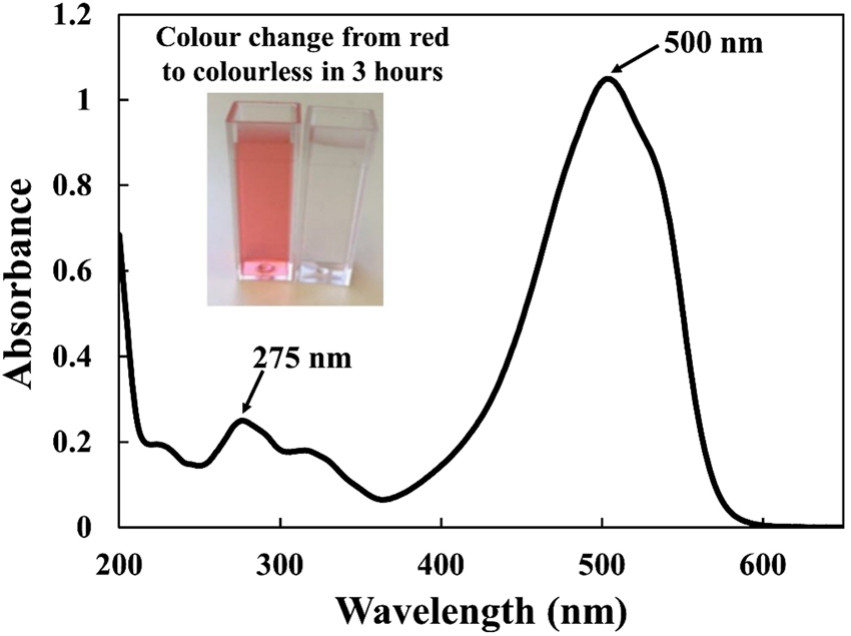
UV-visible spectrum of a methyl orange aqueous solution indicating two absorption maxima. The inset illustrates the methyl orange solution colour change by photocatalysis before and after 3 hours UV exposure over TiO_2_-MFC composite.

The photocatalytic activity of water resistant, thin and flexible cellulose/PAE/titanium dioxide (TiO_2_) nanoparticles (NPs) composites was investigated by following the UV induced degradation kinetics of Methyl Orange (MO) dye aqueous solutions. Composites varying in TiO_2_ NPs and PAE contents were prepared. The UV-vis spectroscopy for MO aqueous solutions denotes two distinct absorption peaks appearing at 275 nm and 500 nm (Fig. 1). The band at 500 nm was selected to measure the effect of photocatalysis on the degradation of MO as this peak decreases rapidly as increases UV light exposure time of the photocatalytic material (Supplementary Fig. S1). In 3 hours, the colour of the MO solution changes from an intense red/orange to colourless, which indicates degradation of MO (Fig. 1 inset).

### Photocatalytic activity performance

**Figure 2:**
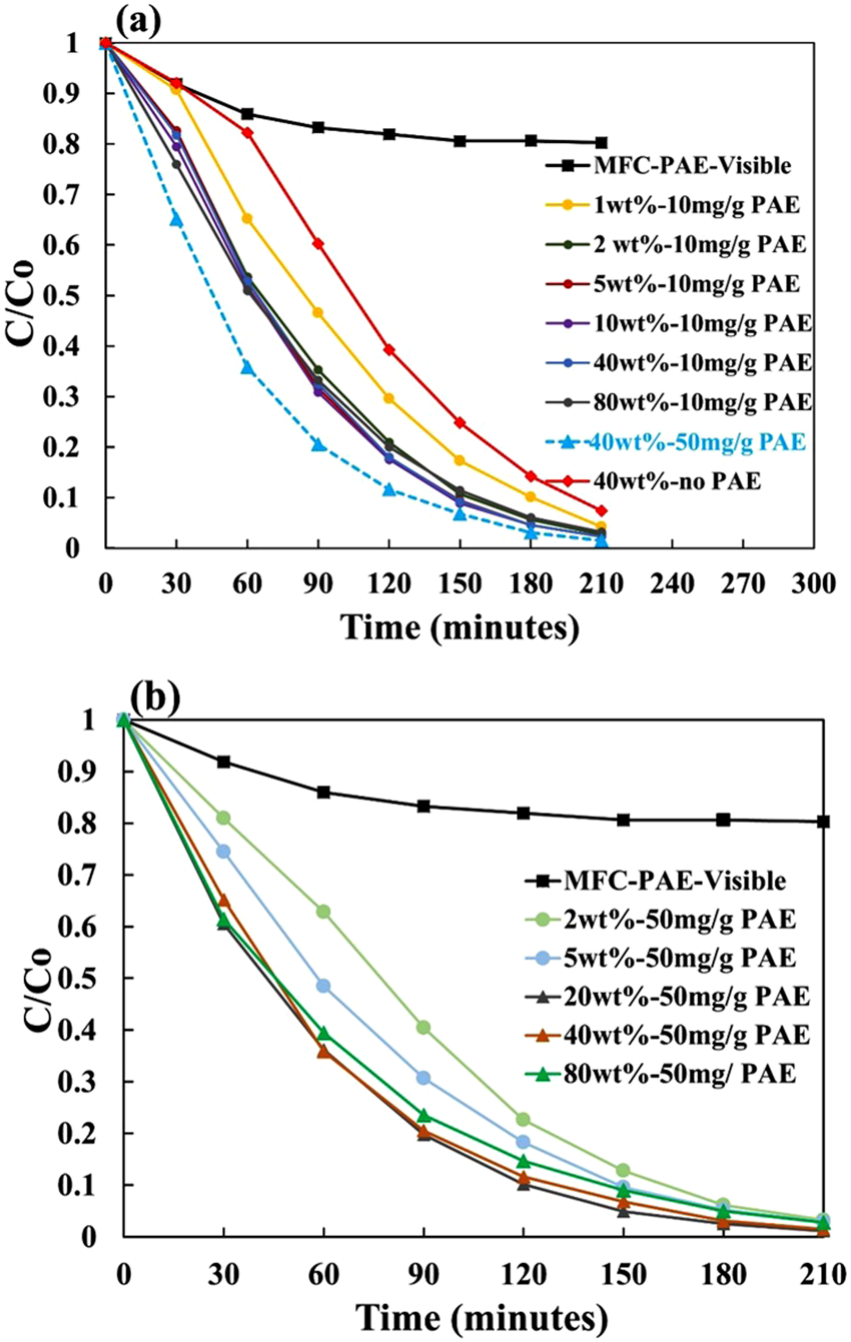
Photocatalytic activity of TiO_2_-MFC composites with different TiO_2_ loadings retained with (**a**) 10 mg PAE/g MFC and (**b**) 50 mg PAE/g MFC.

The photocatalytic degradation kinetics of MO is shown as a function of TiO_2_ loading for 10 and 50 mg PAE/g MFC in Fig. 2a,b, respectively. The intensity of the MO peak decreases with exposure time for all composites. Although the rate at which MO decomposes changes for different composites, it takes roughly 3 hours for MO colour to change from the original red/orange colour to transparent and colourless (Fig. 1 inset).

There is virtually no photocatalytic activity shown by composites without TiO_2_ (MFC-PAE only) as indicated in Fig. 2 (filled squares). Due to the initial adsorption of the MO dye onto MFC, its concentration initially decreased by ~20% in about 2 hours as MFC-PAE paper is kept under visible light. The adsorption equilibrium is gradually reached after 4 hours. Therefore, all test samples were kept in MO solutions and in the dark for 2 hours prior to UV light irradiation.

The performance of the TiO_2_-MFC composites made with lower PAE dosage (10 mg/g) is shown in Fig. 2a. As TiO_2_ loading increased to 1 and 2 wt%, the photocatalytic activity systematically increased. At 2 wt%, a noticeable increase in photocatalytic activity was observed compared to 1 wt%. Composites with addition levels of 5 to 80 wt% TiO_2_ behaved similarly to 2 wt% and showed the highest photocatalytic activity for this group. Photocatalytic activity decreased in a weak exponential fashion to 20% of the original dye concentration after ~120 minutes for the 2–80 wt% TiO_2_ composites group and ~150 minutes for the 1 wt% TiO_2_ composite.

The degradation curve for 40 wt% TiO_2_-MFC composite with no PAE is also shown in Fig. 2a (filled diamonds). There is still photocatalytic activity; this suggests that some TiO_2_ NPs are retained- even with no PAE present.

The performance of TiO_2_-MFC composites with the high PAE dosage (50 mg/g) is shown in Fig. 2b. The degradation pattern is similar to that of composites with 10 mg/g; however, the photocatalytic activity saturation is reached at 20 wt% TiO_2_ loading and remains constant thereafter. The overall degradation rate with 20–80 wt% TiO_2_ was faster than for composites made with 10 mg/g (indicated by plotting the degradation graph for 40 wt% TiO_2_ sheet with 50 mg/g in Fig. 2a-dotted line). Here, the photocatalytic activity decreased to 20% of the original dye concentration after ~90 minutes for the 20–80 wt% TiO_2_ composites group and ~120 minutes for the 2–5 wt% TiO_2_ composite group.

### Photocatalysis repeatability

**Figure 3:**
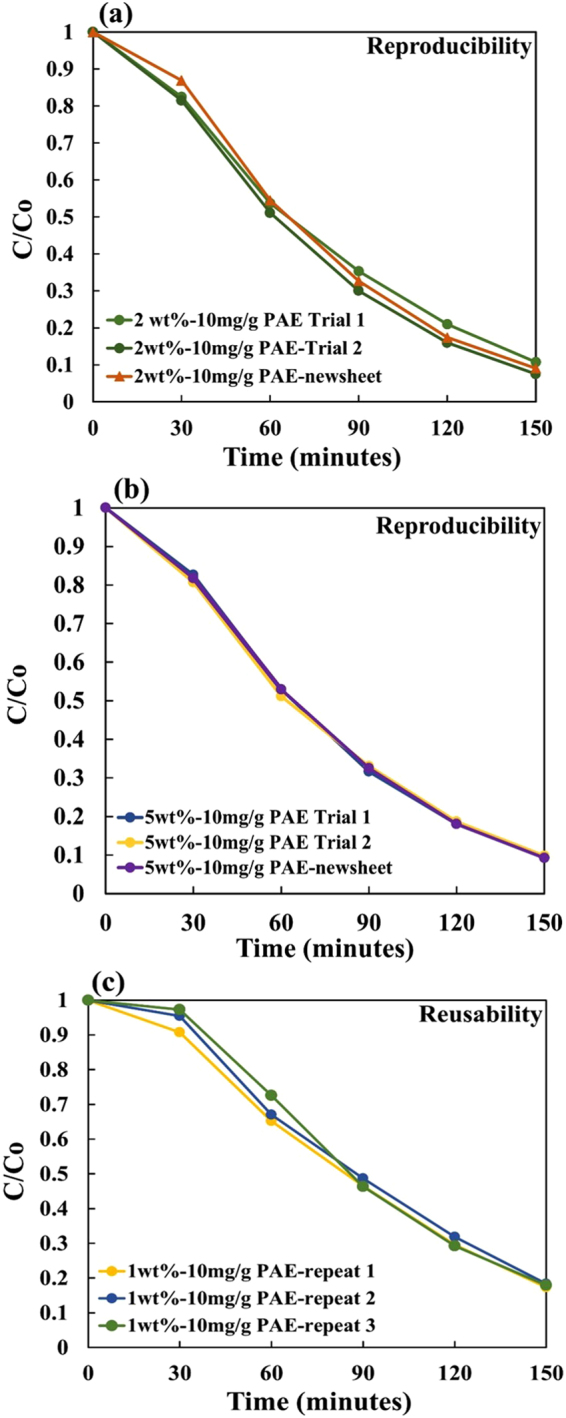
Photocatalytic repeatability and reproducibility for MO solution degradation over UV irradiated MFC-TiO_2_ composites made with 10 mg PAE/g MFC and: (**a**) 2 wt% and (**b**) 5 wt% TiO_2_; (**c**) Composite reusability for 3 full testing cycles (1 wt% TiO_2_ and 10 mg PAE/g).

Uniformity of the sheet and photocatalytic activity repeatability was measured by cutting two test strips (2.5 cm × 2.5 cm) from two different locations from the same original composite. Reproducibility of sheet was tested by preparing two different composite sheets with the same TiO_2_, MFC and PAE content and testing their photocatalytic activity. Figure 3a,b show an excellent repeatability and reproducibility in the MO photocatalytic degradation from composite sheets made with 2 wt% and 5 wt% TiO_2_ retained with 10 mg PAE /g.

Sheet reusability was measured by testing the photocatalytic activity of the same sample 3 times. After each run, the test piece was washed with deionized water to remove any MO residue and dried. The photocatalytic activity of the composite sheets with 1 wt% TiO_2_ and PAE 10 mg/g remains identical even after 3 cycles (Fig. 3c).

### PAE effect on MFC flocculation

**Figure 4:**
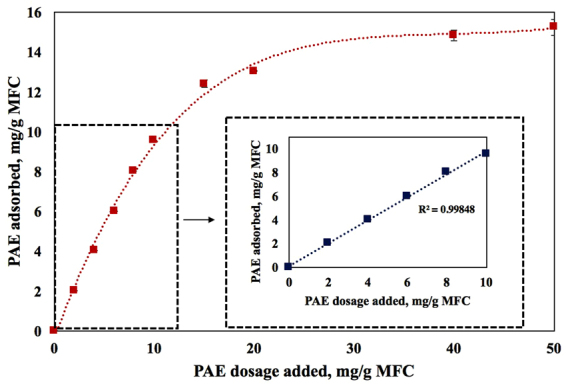
Adsorption isotherm of PAE adsorbed on MFC as a function of PAE concentration in solution. The inset highlights the linear adsorption portion at low PAE concentrations. Error bars indicate the standard deviation.

The adsorption isotherm of PAE onto MFC is presented in Fig. 4. PAE has a high affinity for MFC as shown by the initial linear portion of the curve having a slope of 1 (Fig. 4 inset). The linear part of the curve states that all PAE in solution adsorbs onto MFC until a concentration of 10 mg/g. The PAE adsorption then slows down, to eventually reach a plateau at around 15 mg PAE/g MFC. The kinetics of polyelectrolyte adsorption and the mechanism of inorganic filler coagulation rate as a function of polyelectrolyte is studied in the past^47,48^.

**Figure 5:**
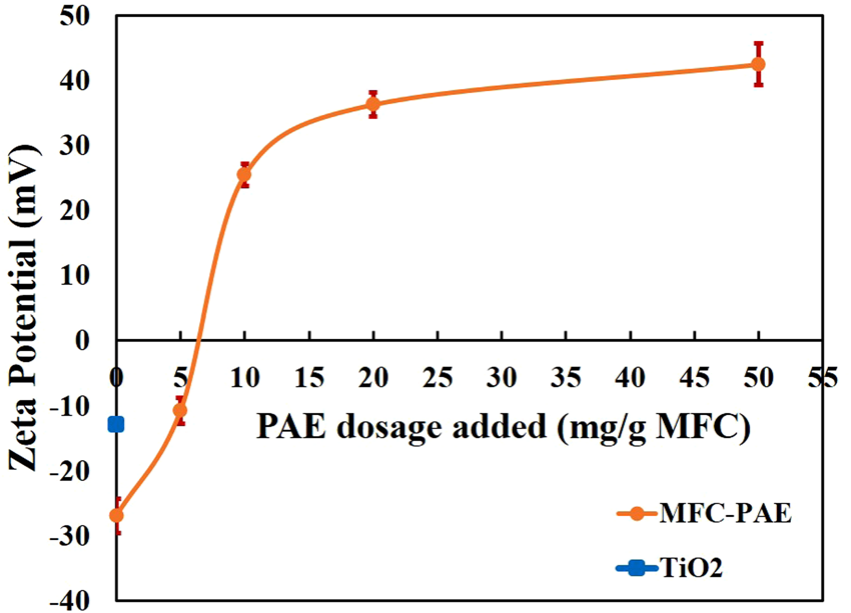
Zeta potential of PAE-MFC suspensions as a function of PAE dosage (mg PAE/g MFC). The supernatant of PAE in a MFC suspension was analyzed after centrifugation according to the reported work of Garnier *et al*.^57^.

Figure 5 shows the MFC zeta potential as a function of PAE concentration. MFC has a zeta potential of −26 mV, while that of TiO_2_ is −11 mV (shown by filled square). MFC charge increases linearly with PAE concentration up to 10 mg/g, corresponding to a charge of +25 mV, to level off thereafter and reach a plateau at +40 mV for a PAE dosage of 50 mg/g. Colloids having an absolute charge higher than 25 mV are considered to be stable. This means that MFC is expected to be stable in solution, while there is a possibility for TiO_2_ to form some small or weak aggregates in solution, and even to weakly deposit onto MFC. However, MFC and TiO_2_ NPs fully covered by PAE are expected to be strongly electrostatically stabilized; no TiO_2_ aggregates nor TiO_2_ adsorption onto MFC are expected.

### Retention efficiency of TiO_2_ in the composites

**Figure 6:**
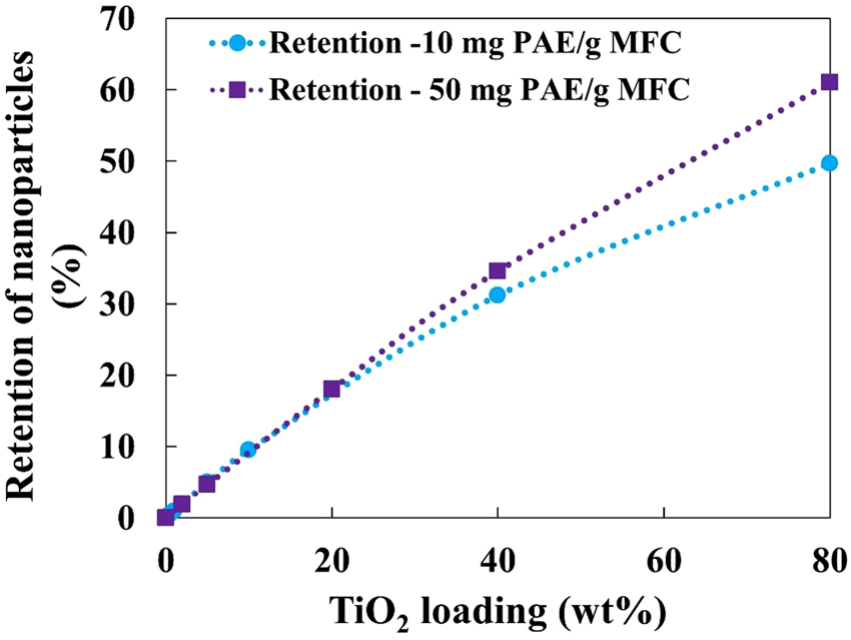
Retention of TiO_2_ nanoparticles as a function of initial TiO_2_ loading for different dosages of PAE (10 mg/g and 50 mg/g).

Retention efficiency is defined as the actual TiO_2_ NPs present in the composite sheet over the total amount used. Retention was measured from mass balance during composite preparation. Figure 6 shows the actual TiO_2_ NPs retention in the composite plotted with respect to NP loading. The retention of NPs increases linearly up to 30 wt% NPs for both PAE dosages. A drop in the retention efficiency for both PAE dosage is observed as increases NPs loading. Afterwards the retention of NPs for 10 mg/g PAE drops faster than for 50 mg/g PAE. For 80 wt% NP loading, the composite with 10 mg PAE /g retains 50% of the NPs, while that with 50 mg PAE /g retain 60% NPs.

### TiO_2_ and MFC morphology in composites

**Figure 7:**
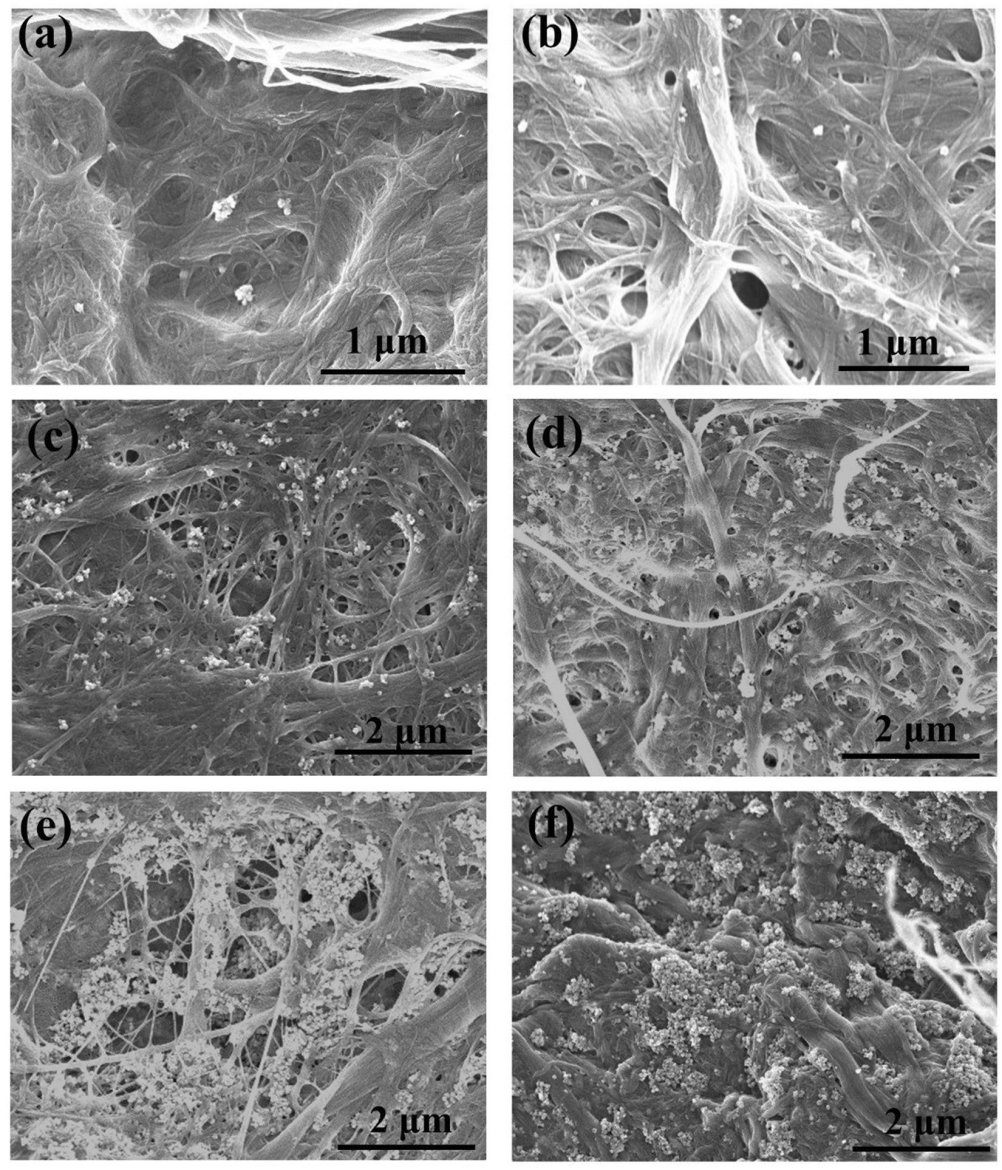
SEM images of TiO_2_ composites with 10 mg PAE/g MFC: (**a**) 1 wt%, (**b**) 2 wt%, (**c**) 5 wt%, (**d**) 10 wt%, (**e**) 40 wt% and (**f**) 80 wt% TiO_2_.

**Figure 8:**
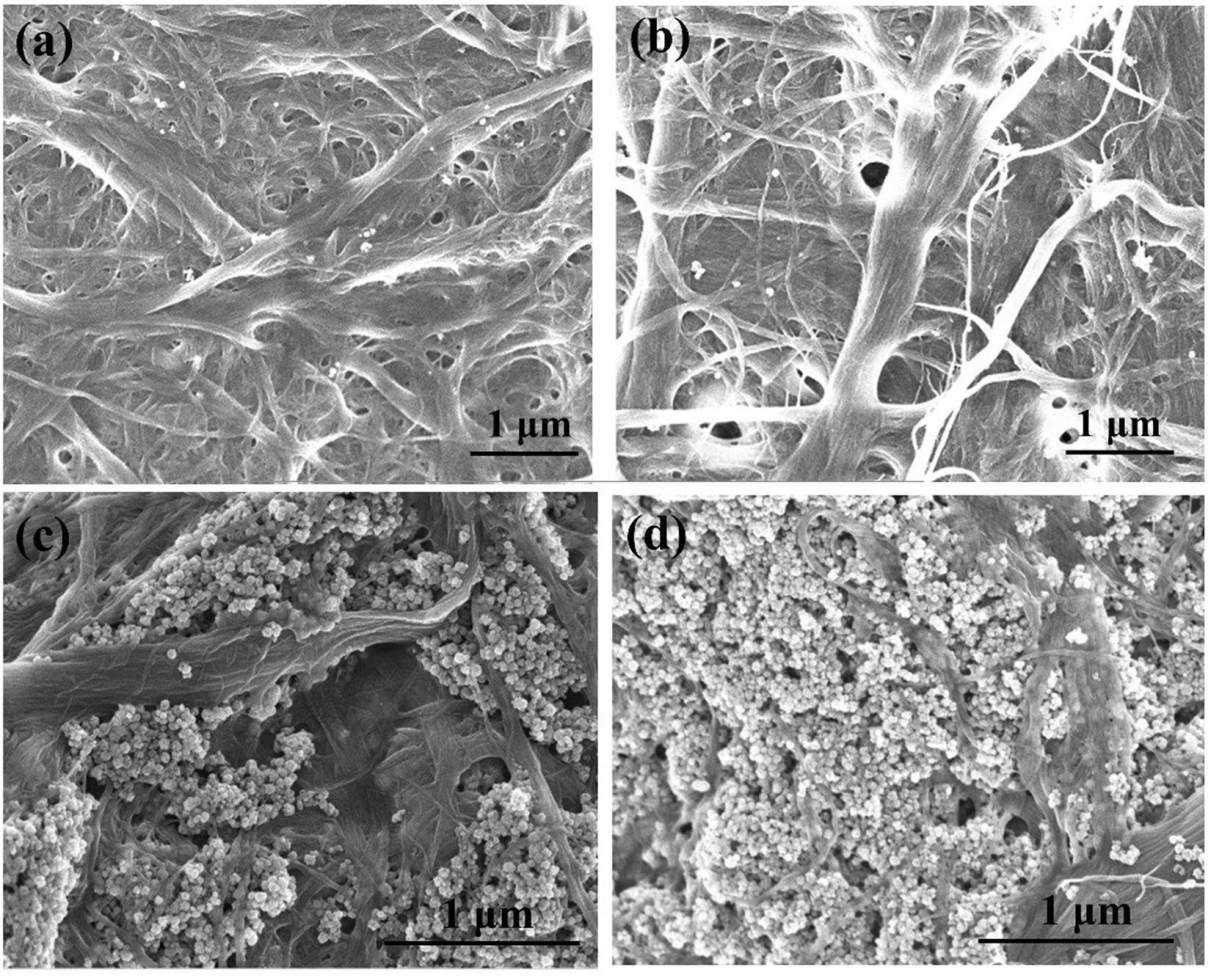
SEM images of TiO_2_ composites with 50 mg PAE/g MFC: (**a**) 2 wt%, (**b**) 5 wt%, (**c**) 20 wt%, and (**d**) 80 wt% TiO_2_.

Scanning electron microscopy (SEM) was performed on all MFC/TiO_2_ composites with PAE dosage of 10 mg/g and 50 mg/g and different TiO_2_ NP’s content (Figs 7 and 8). For 10 mg PAE/g, individual NPs or very small TiO_2_ aggregate are present on the composite surface for 1–2 wt% TiO_2_ loadings (Fig. 7a,b). Relatively large TiO_2_ aggregates are observed for 5–80 wt% TiO_2_ loadings (Fig. 7c–f); the NP surface coverage seems to be similar for 40 wt% TiO_2_ loadings and higher.

For composites with 50 mg PAE/g, individual TiO_2_ NPs are seen up to 5 wt% loading (Fig. 8a,b), beyond which NPs aggregates into big clusters (Fig. 8c,d). Again, the surface coverage of TiO_2_ present at higher loadings (20–80 wt%) all looks identical but higher than those made with the lower PAE dosage (10 mg/g).

Interestingly, TiO_2_ NPs aggregates are present on the surface of MFC fibres rather than in the pores formed between fibres (Figs 7 and 8). This is due to the preparation method, where PAE is added first to MFC to create a PAE monolayer on MFC, followed by TiO_2_ addition onto the MFC-PAE suspensions. The aggregates are irregular in shape and size. The MFC fibre structure does not change even at high TiO_2_ loading; TiO_2_ NPs do not accommodate themselves in the pores between fibres.

### Small angle X-ray scattering (SAXS)

**Figure 9:**
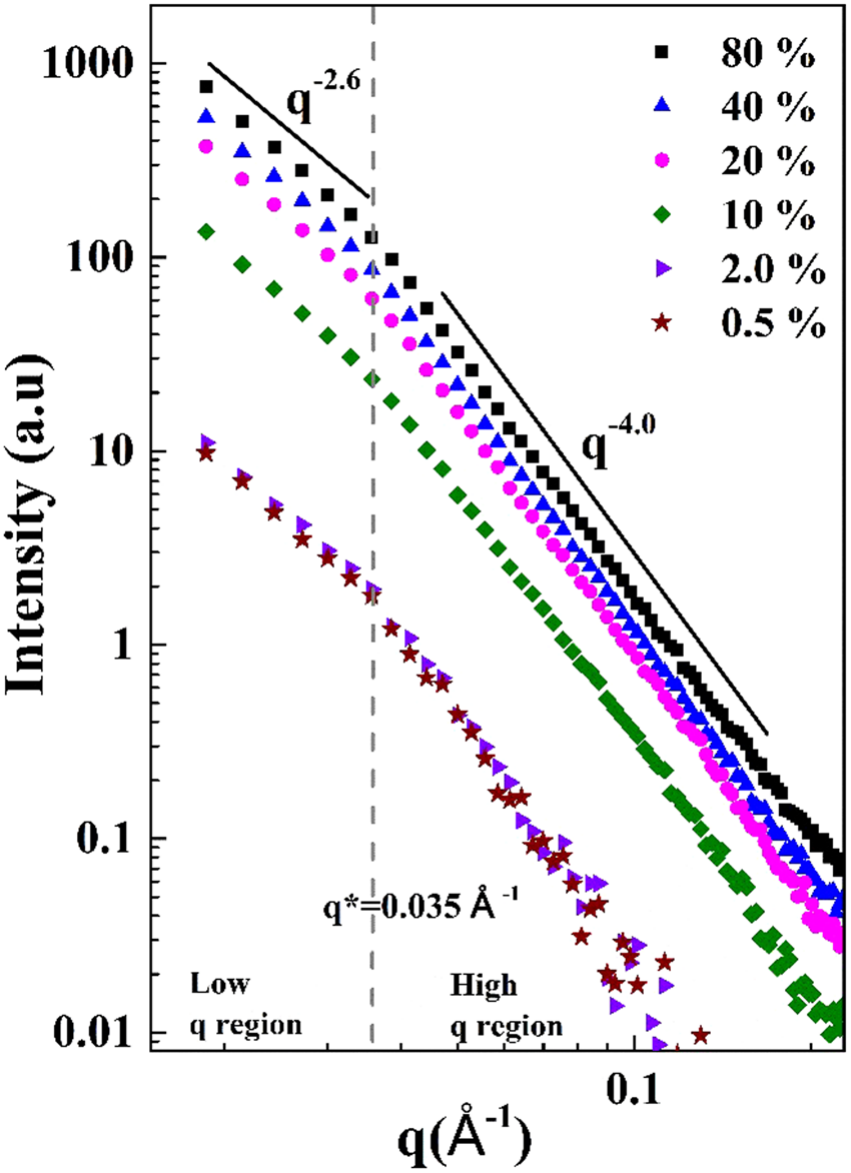
SAXS curves for the MFC/TiO_2_ composites with 10 mg/g of PAE and different loading of NPs from 0.5 to 80 wt%.

Small angle X-ray scattering (SAXS) was performed on all composites to measure the average NPs distribution per unit volume. Figure 9 shows SAXS curves for samples prepared with 10 mg PAE/g and different TiO_2_ loading (0.5–80 wt%). The SAXS curves intensity increases with the concentration of TiO_2_ NPs in the composites. All SAXS curves show a kink at q* = 0.035 Å^−1^ which divides SAXS curves into two different slope regions referred to as low and high *q* region (shown by dashed line).

The slopes in SAXS curves represent the fractal dimensions at different length scales. At high *q* region, the slope represents the surface scattering/fractals (smoothness of surface) and at lower *q* values, the slope shows the scattering from aggregates/mass fractals. Slope varies as the power-law exponent (q^−α^) of scattering intensity. For the mass fractals, value of α lies within 0 < α < 3, and 3 < α < 4 for surface fractals^49,50^.

In Fig. 9, the slope in the high *q* region is q^−4^ (q* > q) which shows the surface fractal region while α = 4 reveals that the NPs surface is smooth. The slope for the low *q* region is q^−2.6^ (q* < q) and represents the mass fractal region (α = 2.6) which is interpreted as evidence of the formation of NPs aggregates^51^.

## Discussion

### Quality and durability of MFC-PAE-TiO_2_ composites

TiO_2_ paper composites of different TiO_2_ nanoparticles (NPs) content were prepared with two PAE dosages (10 mg and 50 mg PAE/g MFC). The photocatalytic activity was tested under standard conditions using aqueous solutions of methyl orange (MO) dye. MO is an azo dye of relatively high toxicity and poor biodegradability which provides a good reference for waste water residues from the printing and dying industries^26^. The effect of TiO_2_ NPs (30–50 nm) and their aggregates distribution as influenced by PAE was analysed by combining scanning electron microscopy (SEM), small angle X-ray scattering (SAXS) and photocatalytic kinetics. NPs aggregation is crucial as it influences the ability of the material to absorb and scatter incoming radiation, which greatly affects the photocatalytic activity^52^.

Here, the addition of PAE was part of the strategy to engineer paper wet strength to develop durable MFC-TiO_2_ composites able to sustain harsh applications in aqueous environments. PAE serves two functions. First, it cross-links cellulosic fibres, producing non-woven materials that remain durable when used wet and under long UV light exposure; second, it retains TiO_2_ NPs onto the MFC fibres and within the fibrous composite structure. Previous work done with PAE in cellulose paper systems has proven that an addition of 10 mg PAE/g fibre retain the wet-strength of the paper, making it water resistant^42^. MFC-PAE-TiO_2_ composites are very efficient at degrading organic dye in solution and have the sufficient wet strength to be robustly manipulated. The photocatalysis results in Fig. 2 also indicates that neither PAE nor MFC contributes to the photocatalytic activity which is solely based on the presence and distribution of TiO_2_.

The flexible TiO_2_ composite sheets investigated are simpler to produce than most methods described in literature that use dopants, carbon and other materials and tedious preparation methods^26,27,33,53^. The composites show excellent photocatalytic activity in degrading MO and are uniform, reproducible and re-usable (Fig. 3). In the reusability test cycles, no noticeable mass loss of TiO_2_ NPs or broken MFC structure was observed. The composites can easily be removed from the polluted water after the reaction is completed and are expected to be fully recyclable using current equipment and processes^42^. Figure 3c shows that the composites produced are reusable even after the third cycle.

TiO_2_ has a characteristic UV-Vis absorbance peak which depends on particle size and concentration. Anatase TiO_2_ absorbance occurs at wavelength range of 350–390 nm^54^. MO solutions were monitored through UV-Vis every 30 minutes. In this time interval, no characteristic peak between 350–390 nm was observed (Supplementary Fig. S1). This indicates that no TiO_2_ NPs have been desorbed and diffused from the composite into the MO solution which confirms the stability of TiO_2_ in the MFC matrix. Using PAE to embed TiO_2_ into the MFC matrix and consolidate the composite resolves the instability problems that have typically arisen from NPs alone.

### Effect of PAE on TiO_2_ nanoparticle retention

We raised two hypotheses in this study. The first is that the distribution and aggregation of TiO_2_ NPs both affect the TiO_2_-MFC composite photocatalytic activity; the second is that PAE dosage governs the retention of TiO_2_ NPs. PAE adsorption onto MFC reaches the maximum capacity of adsorption at 15 mg/g (Fig. 4). This is about twice the value reported for PAE adsorption onto eucalyptus fibres (8.6 mg/g)^55^. Assuming MFC to be uniform cylinders of 10.37 μm long and of average diameter 73 nm^56^, the specific surface area of MFC is 36.5 m^2^/g. The surface area reported for MFC characterized through mercury porosimetry and BET are 31.1 m^2^/g^57^ and 35 m^2^/g^58^, respectively. A specific PAE adsorption of 0.41 mg/m^2^ results for MFC which is consistent with the range of polyelectrolyte adsorption (0.4–1 mg/m^2^)^59^.

Because of its low molecular weight (200 kDa) and chemical composition with 2 interacting functionalities (primary and secondary amines and azetidinium), PAE is expected to transfer to some extent from MFC to TiO_2_ upon collision^60^. This would result in TiO_2_ aggregate formation. Such TiO_2_ aggregates can be seen by SEM, especially at the high TiO_2_ loadings (Figs 7 and 8).

Adding 10 mg PAE/g saturates all MFC fibres which induces a charge reversal to +25 mV—charge of opposite sign but equivalent magnitude to the original (Fig. 5). At this dosage, all PAE is adsorbed onto MFC (Fig. 4). No excess free PAE is expected in solution. Under those conditions, all TiO_2_ NPs are anticipated to adsorb onto MFC with a high retention efficiency. This means that the TiO_2_ content on MFC fibres should increase pseudo linearly with TiO_2_ add-on.

At 50 mg PAE/g, all the MFC fibres are saturated with PAE and there is an important excess free PAE remaining in solution. PAE adsorbs at 15 mg/g MFC; this means 18 mg is consumed by MFC fibres, leaving 42 mg PAE in solution. Assuming PAE adsorbs onto TiO_2_ in the same morphology/conformation as on MFC, at 0.41 mg/m^2^ (specific PAE adsorption on MFC), then the free PAE in solution can cover 102 m^2^ of TiO_2_, or nearly 2.88 g of TiO_2_ which corresponds to 67 wt% loading in the composites. This means for TiO_2_ content lower than 67 wt%, all TiO_2_ NPs are expected to be fully covered by PAE, as are the MFC fibres to which PAE was previously adsorbed; no TiO_2_ retention due to electrostatic interactions is expected.

Figure 5 shows the zeta potential of +40 mV at 50 mg PAE/g of suspension. This reveals a strongly electrostatically stabilised system, and no adsorption of TiO_2_ onto MFC, or homocoagulation of TiO_2_ or MFC fibres are expected. That was not the case. SEM (Fig. 8) and photocatalysis activity (Fig. 2b) contradict this expectation. For one, TiO_2_ NPs and aggregates are seen by SEM to be present on MFC surfaces. Also, photocatalysis is at the highest for composites containing 20 to 80 wt% TiO_2_. Further, there is photocatalysis and TiO_2_ retention even for the very low loadings (2 to 5 wt% TiO_2_) for which not only are both components of the system saturated with PAE, but there is also a large excess of PAE in solution. These results state that PAE does not follow trivial polyelectrolyte adsorption behaviour. PAE very likely adsorbs as partial multilayer at very high concentrations. Adsorption isotherm was thus further quantified under the exceptional conditions of 120 mg PAE/g MFC. A slight increase in adsorption capacity was indeed recorded (Supplementary Fig. S2). PAE is known for its ability to self-cross link during drying, which suggests some ability to assemble at very high concentrations.

The PAE adsorption, TiO_2_ retention and TiO_2_ coagulation expectations from fundamental principles clearly contradict the photocatalytic, retention efficiency measurements as well as the SEM results. This means that PAE behaves differently from the trivial polyelectrolyte adsorption behaviour previously discussed.

### Composites photocatalysis activity

Photocatalysis is a surface phenomenon. Only the TiO_2_ NPs retained on the composite external surface, which is irradiated by UV light, can take part in the photocatalysis process. At 10 mg PAE/g, all PAE is adsorbed onto MFC (Fig. 4) creating a monolayer of PAE on MFC. Since maximum adsorption results at 15 mg/g, this means that at 10 mg PAE/g some of the MFC surface is still not coated by PAE. As TiO_2_ NPs are added, the amount of TiO_2_ that can be retained on the surface for 10 mg of PAE is less than at 50 mg PAE. This can be seen by the higher retention found for the 50 mg PAE/g as compared to those made with 10 mg PAE/g (Fig. 6).

However, at 50 mg PAE/g, the entire MFC surface is completely covered by PAE and there is excess in solution. This excess PAE interacts firstly with the incoming TiO_2_ NPs and hinders their agglomeration into large agglomerates by electro-steric stabilisation. This increases the TiO_2_ surface area available for photocatalysis; this also accounts for the higher photocatalysis for composites made with 50 mg PAE/g.

Saturation of the photocatalytic activity after reaching a critical TiO_2_ loading (at a particular PAE dosage) might be due to the formation of large agglomerates which constrain the effective surface area available for photocatalysis. SEM showed (Fig. 7) that at 2 wt% TiO_2_ (10 PAE mg/g), there are less individual NPs compared to composites with 40–80 wt% TiO_2_.

The influence of wet strength resins on photocatalytic activity was first studied by Zhang *et al*. (2013) who claimed that PAE addition slightly decreased photocatalytic activity due to a reduction in TiO_2_ retention in paper^33^. This statement contradicts our results. We found that PAE helps retain more TiO_2_ NPs in paper (Fig. 6) which is in agreement with the SAXS results (Fig. 8). However, comparison of photocatalytic activity results is not direct nor straightforward; many variables influencing catalytic activity. This is illustrated in Table 1 which compares the photocatalytic dye degradation in aqueous solution from selected studies with TiO_2_/cellulose composites.

**Table 1:** Literature comparison of the material performances.

Composite type	Degrading medium	UV lamp conditions	Time taken to degrade by 90%	Reference
40 wt% TiO_2_ nanobelt paper	Methyl Orange	30 W, 294 nm	~2.5 hours	^37^
TiO_2_ particle size: 21 nm	20 mL			
Test piece: 1 × 1 cm^2^	0.02 g/L			
TiO_2_/cellulose fibre composite	Methyl Orange	30 W	~7 hours	^33^
TiO_2_ particle size: 25–30 nm	20 mL			
Test piece: 3 × 3 cm^2^	20 mg/L			
TiO_2_/regenerated cellulose paper	Phenol	6 W, 253 nm,	~102 hours	^63^
Test piece: 1 × 8 cm^2^	320 mL			
	67.2 mg/L			
10 wt% TiO_2_/bleached softwood cellulose fibre composite	Methyl Orange	72 W	~13 hours	^64^
Test piece: 2.5 × 0.7 cm^2^	0.25 mM in 4 ml water	320–400 nm		
TiO_2_ nanorods/regenerated cellulose films	Methylene blue	30 W, 312 nm	~4 hours	^31^
	150 mL			
	40 mg/L			

Note: the independent variable is radiation intensity: photon per unit area per time. Geometry of the system, particularly the distance from surface and the diffusion angle, affects this a lot.

**Figure 10:**
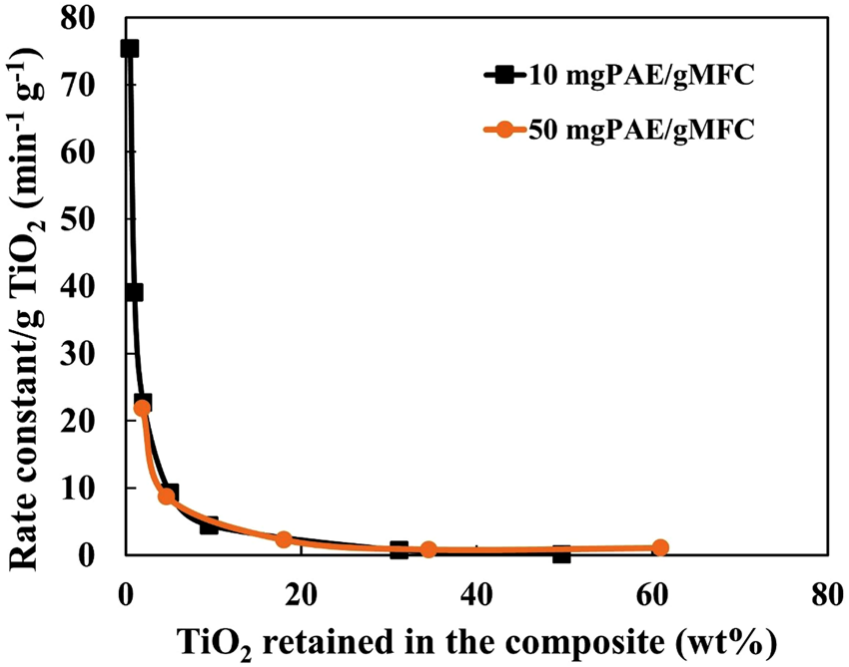
Photocatalytic performance of the material represented by plotting the variation in rate constant as function of the TiO_2_ amount retained in the composite.

Figure 10 shows the rate constant normalized per grams of TiO_2_ NPs present in the composite samples tested in this study. The graph indicates that low TiO_2_ loadings provide the best photocatalytic activity per unit TiO_2_. This material performed best at 0.5–2 wt% TiO_2_ loading. The photocatalytic activity of the TiO_2_-MFC composites prepared at this work is very effective under UV irradiation; only a small amount of TiO_2_ is needed to effectively degrade MO.

TiO_2_-cellulose composites were engineered to be easy to manufacture by process straightforwardly scalable; the composites produced are water resistant, flexible, cost effective, and most importantly, reproducible. These composites are green and can be used in applications such as waste water treatment, antibacterial, drug delivery and medical^61,62^.

## Conclusion

Water resistant microfibrillated cellulose (MFC)—polyamide-amine-epichlorohydrin (PAE)—titanium dioxide (TiO_2_) composites were prepared by a simple two-step process, where PAE was first added to a MFC suspension, followed by TiO_2_ addition. These composites are simple to prepare, economical and the process is easily scalable. Photocatalytic activity of the composites produced was tested by following the degradation of methyl orange (MO) aqueous solutions under UV irradiation. Results show that neither MFC nor PAE or their combination contributed to photocatalytic activity; only the TiO_2_ nanoparticles (NPs) embedded in the sheets do. TiO_2_ NPs are uniformly distributed within the composite sheets as shown by the excellent special repeatability in photocatalysis measured. Further, these composites are reusable; the same reproducible photocatalytic efficiency was achieved by testing a same test strip 3 times with no loss of TiO_2_ NPs leaching into solution.

Comparing photocatalytic activity of composites with two different dosage of PAE (10 and 50 mg PAE/g of MFC) revealed a higher activity and TiO_2_ NPs retention for the high PAE dosage. MO degraded to 5% of its original concentration in 180 min for composites with low PAE and 150 min for composites with high PAE. Photocatalytic is a non-monotonous function of TiO_2_ content. For composites made with 10 and 50 mg PAE/g and various amounts of NP, the photocatalytic activity increased up to 2 and 20 wt% TiO_2_ NP and remained constant thereafter. SEM indicated that at low TiO_2_ loading, NPs retain as individual particles on MFC, whereas TiO_2_ aggregates at higher loadings. SAXS showed the formation of mass fractals aggregates at different NPs loading. PAE adsorption isotherms revealed a maximum PAE adsorption on MFC (Γ_max_) at 15 mg/g. Expectation resulting from PAE steric and bridging mechanism with maximum coagulation at half surface coverage contradicted the TiO_2_ retention efficiency measurements. This suggests that PAE does not follow trivial polyelectrolyte adsorption behaviour. The current study provides a novel insight in engineering NPs embedded cellulose based biodegradable, flexible and recyclable composites with high potential for applications requiring photocatalysis without any residual contamination.

## Experimental

### Materials

Microfibrillated cellulose (MFC) was purchased from DAICEL Chemical Industries Limited, Japan (grade Celish KY-100G). MFC was supplied at 25 wt% solids and stored at 5^o^C as received. The mean diameter and the aspect ratio of MFC was 73 nm and 100–150, respectively^56^. Anatase titanium dioxide (TiO_2_) was purchased from US Research Nanomaterials, USA. The nanoparticle (NP) size ranged within 30–50 nm and was received at 40 wt% solids. The commercial polyamide-amine-epichlorohydrin (PAE) was provided by Nopco Paper Technology Pty Ltd, Australia (33 wt% solids). Methyl Orange (MO) was purchased as a powder from Sigma Aldrich.

### Methods

#### MFC sheet preparation

MFC sheets were prepared using a standard British hand sheet maker (model T205). The hand sheet maker was equipped with a woven filter with an average opening of 74 microns. A Whatman wet strengthened filter paper (WHAT1114-185) with a pore size of 25 microns was placed on top of the woven filter and a 0.3 wt% MFC suspension (with 1.2 g dry mass of MFC) was poured into the column. Once the water drained under gravity, the wet film was taken out using blotter papers, the filter paper was removed, and the sheet was pressed at 385 kPa for 5 minutes and then dried at 105 °C using a sheet drier.

#### MFC-PAE-TiO_2_ composite sheet preparation

Two sets of MFC-PAE-TiO_2_ composites were produced:

1. Composites with low PAE dosage: 0.3 wt% MFC (1.2 g fixed), 0.01 wt% PAE (10 mg PAE/g MFC fixed) and with varying TiO_2_ loading at 0.5, 1, 2, 5, 10, 40 and 80 wt%.
2. Composites with high PAE dosage: 0.3 wt% MFC (1.2 g fixed), 0.03 wt% PAE (50 mg PAE/g MFC fixed) and with varying TiO_2_ loading at 2, 5, 20, 40 and 80 wt%.

The composites were prepared in a two-step process. Firstly, the PAE suspension was added at a constant flowrate of 30 mL/min into a beaker containing 0.3 wt% (1.2 g fixed) MFC, while stirring the suspension using a hand stirrer (high shear mixing). Secondly, 0.1 wt% TiO_2_ suspensions were strongly sonicated (sonicator model: VCX750 purchased from John Morris Scientific Pty Ltd for, used for 10 minutes at 80% amplitude) and added to the MFC-PAE suspension at a constant flowrate of 20 mL/min, while stirring the entire suspension.

Suspensions were poured into the British hand sheet maker and the composite sheets were made as described above.

#### Photo-degradation of methyl orange

**Figure 11:**
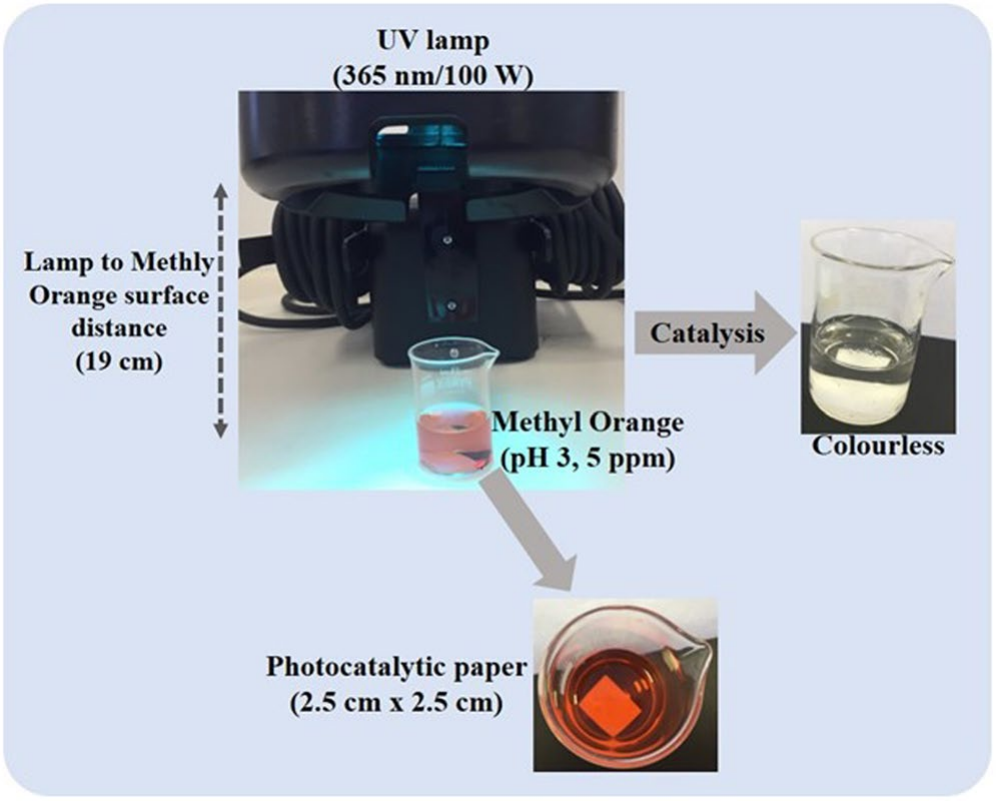
Experimental setup for photocatalytic degradation of methyl orange. The paper samples were cut to squares (2.5 cm × 2.5 cm).

The photocatalytic degradation tests were carried out at room temperature using MO as a model dye. A 100 W lamp (365 nm wavelength) was used as the light source. The samples were cut into 2.5 cm × 2.5 cm pieces and dispersed in a 50 mL beaker filled with 15 mL of 5 ppm pH 3 MO aqueous solution. The sample immersed in the beaker was kept in the dark for 2 hours until the maximum adsorption of MO by MFC prior to photocatalytic experiment was reached. The beaker was then subjected to UV radiation for MO photocatalytic degradation. The distance between the liquid surface and the light source was 19 cm. At given irradiation time intervals, the solution was collected for analysis using UV-vis spectroscopy (Model: Cary 60 UV-Vis, Agilent Technologies). Experimental set up is shown in Fig. 11.

### Characterization

#### Structure and morphology study

Scanning electron microscopy (SEM) analysis of the composite sheets was performed using a FEI Magellan 400 FEGSEM. Samples were cut into 3 mm × 3 mm mounted onto a metal sample holder and coated with a thin layer of Iridium prior to imaging.

#### Particle and colloid charge

The zeta potential measurements of MFC, TiO_2_ and PAE were performed with a Nanobrook Omni (Brookhaven Instruments) in a cuvette cell at 25 °C. The zeta potential was calculated, using the supplied software, by determining electrophoretic mobility from an electrophoresis experiment using laser Doppler velocimetry and applying the Smoluchowski equation. PAE at 0.01 wt% concentration was added at different dosages to a 0.3 wt% MFC suspension and mixed using a hand stirrer for 3 minutes. MFC-PAE suspension was centrifuged at 4400 rpm for 20 minutes to remove big agglomerates and the supernatant was used to measure zeta potential.

#### PAE adsorption on MFC

This method is adopted, as described by Peng and Garnier^48^. Particle charge detector (Mutek PCD-03, BGT Instruments) was used to titrate the amount of PAE in the supernatant after centrifugation using an opposite charged polyelectrolyte until point of zero charge is met. The polyelectrolyte used was PES-Na of known concentration (0.000125 N), and was added at 10 mL dosages to the PAE-MFC supernatant. Titrant consumption was measured in mL and converted into PAE concentration through a standard curve method.

#### Small angle X-ray scattering (SAXS)

SAXS measurements were made on a Laboratory Bruker N8 Horizon using a CuKα (λ = 0.154 nm) micro-source. The sample to detector distance was 0.6 m covering the q range between ∼0.15 to 3.7 nm^−1^. The scattered photons after interacting with the sample were collected using a 2D Vantec-500 detector (pixel size ∼70 μm × 70 μm). Final scattering curves were obtained after data reduction and radial averaging using Bruker EVA software.

## Electronic supplementary material

Supporting Information
