# Supplementary material for: Water Resistant Cellulose – Titanium Dioxide Composites for Photocatalysis
Source: Sci Rep. 2018 Feb 2;8:2306. doi: 10.1038/s41598-018-20569-w (PMC5797173; doi:10.1038/s41598-018-20569-w)
Supplement: Supplementary file 1 — Supporting Information [file 41598_2018_20569_MOESM1_ESM.doc]

# *Supporting Information*

# Water resistant cellulose – titanium dioxide composites for photocatalysis.

Uthpala Manavi Garusinghe, Vikram Singh Raghuwanshi, Warren Batchelor*, Gil Garnier*

BioResource Processing Research Institute of Australia (BioPRIA), Department of Chemical Engineering, Monash University, Clayton 3800, VIC, Australia.

*for correspondence:

[gil.garnier@monash.edu](mailto:gil.garnier@monash.edu),

warren.batchelor@monash.edu

# Supplementary Information


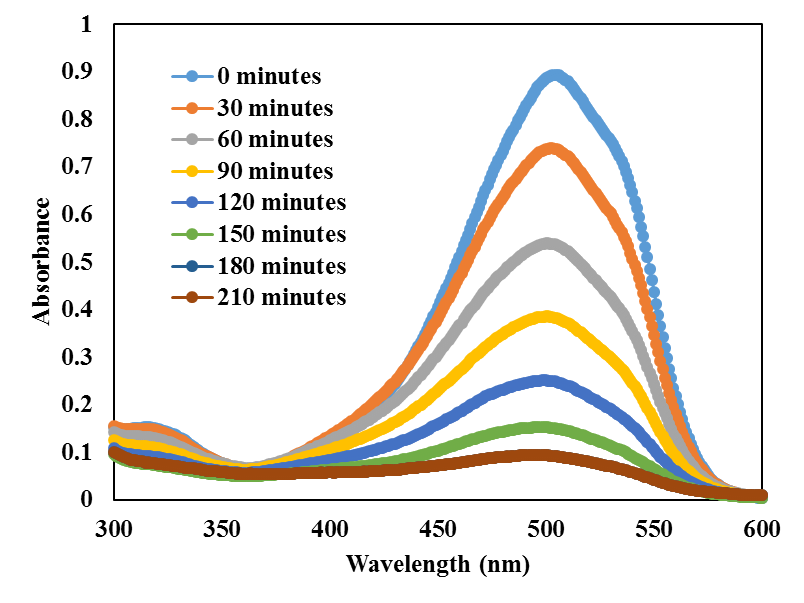


**Fig. S1: Effect of UV exposure time on adsorption spectra for the composite with 1 wt% TiO_2_ loading and with 10 mg PAE/g MFC (second repeat of the same composite test sample).**


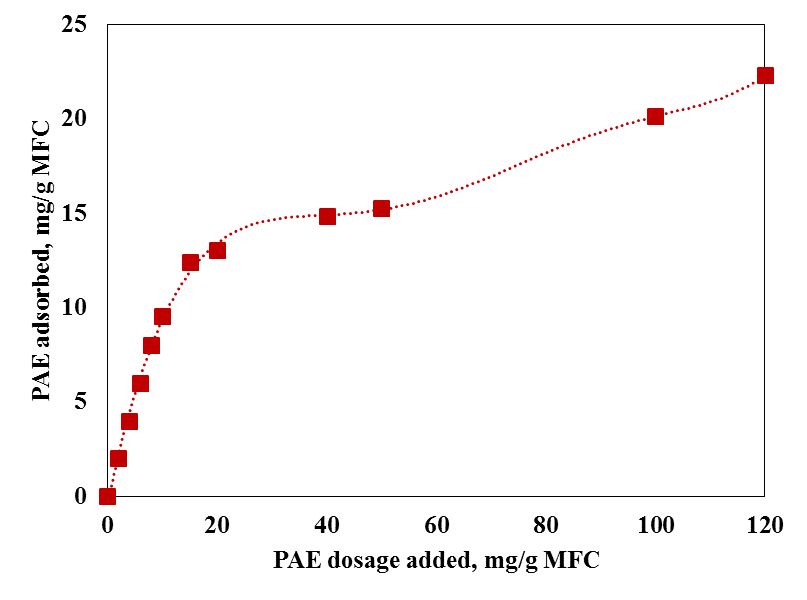


**Fig. S2: PAE Adsorption isotherm on MFC for extreme PAE dosages.**
